# Immune dysregulation in chronic diabetic wounds: therapeutic opportunities for healing reprogramming

**DOI:** 10.3389/fimmu.2026.1889465

**Published:** 2026-08-18

**Authors:** Wenhui Yu, Qiang Li, Jinjun Wang, Jiaming Fu, Yimin Hao, Zhaowei Wang, Heng Xu, Meijun Zhang, Gang Zhao, Yingying Ma

**Affiliations:** 1 Department of Vascular Surgery, The First Affiliated Hospital of Heilongjiang University of Traditional Chinese Medicine, Harbin, Heilongjiang, China; 2 Qingdao Traditional Chinese Medicine Hospital, Qingdao Hiser Hospital Affiliated of Qingdao University, Qingdao, Shandong, China; 3 Department of Vascular Surgery, Heilongjiang University of Chinese Medicine, Harbin, Heilongjiang, China; 4 Department of Vascular Surgery, Heilongjiang Provincial Hospital, Harbin, Heilongjiang, China

**Keywords:** chronic inflammation, diabetic wound healing, M1/M2 macrophages, macrophage polarization, NLRP3 inflammasome, pyroptosis, therapeutic targeting

## Abstract

Diabetic foot ulcers (DFUs) represent a devastating complication of diabetes mellitus, affecting approximately 25% of diabetic patients and contributing significantly to morbidity, mortality, and healthcare costs worldwide. Chronic non-healing wounds in diabetes are characterized by persistent inflammation, impaired immune cell function, and dysregulated cellular responses. The nucleotide-binding oligomerization domain-like receptor family pyrin domain-containing 3 (NLRP3) inflammasome has emerged as a critical mediator of chronic inflammation and wound chronicity in diabetes. This comprehensive review examines the molecular mechanisms linking NLRP3 inflammasome activation, macrophage polarization dynamics, and wound healing impairment in diabetic conditions. We discuss how hyperglycemia-induced oxidative stress, advanced glycation end-products (AGEs), and mitochondrial dysfunction converge to activate the NLRP3 inflammasome, leading to caspase-1 activation, interleukin-1*β* (IL-1*β*) and IL-18 maturation, and gasdermin D (GSDMD)-mediated pyroptosis. The reciprocal relationship between inflammasome activation and macrophage polarization bias toward the pro-inflammatory M1 phenotype is explored, highlighting how this perpetuates a chronic inflammatory wound microenvironment. Additionally, we examine the role of neutrophil extracellular traps (NETs) in amplifying inflammasome-mediated inflammation. Emerging therapeutic strategies targeting the NLRP3 inflammasome pathway are reviewed, including pharmacological inhibitors (MCC950, glyburide, disulfiram), natural compounds (quercetin, resveratrol, tanshinone IIA), and advanced biomaterial-based approaches. The potential for macrophage phenotype reprogramming through metabolic intervention and the integration of inflammasome modulation with tissue engineering strategies are discussed. Understanding the intricate crosstalk between NLRP3 inflammasome activation and macrophage polarization provides a foundation for developing targeted therapies that can reprogram the diabetic wound microenvironment from a chronic inflammatory state toward effective healing resolution.

## 1 Introduction

Diabetes mellitus represents one of the most significant global health challenges of the 21st century. According to the 11th edition of the IDF Diabetes Atlas, approximately one in nine adults worldwide exceeding 500 million individuals was living with diabetes in 2024, a figure projected to rise to nearly 900 million by 2050 (1). Among the numerous complications associated with diabetes, diabetic foot ulcers (DFUs) constitute a particularly devastating manifestation, affecting approximately 25% of diabetic patients during their lifetime (2). DFUs serve as the leading cause of non-traumatic lower extremity amputations and contribute substantially to mortality, with five-year mortality rates following amputation exceeding those of many common malignancies (3).

The pathophysiology of impaired wound healing in diabetes is multifactorial, involving peripheral neuropathy, peripheral arterial disease, and a complex array of cellular and molecular dysfunctions (4). Central to the chronicity of diabetic wounds is a persistent inflammatory state characterized by dysregulated immune cell function, excessive production of pro-inflammatory cytokines, and and impaired progression through the normal phases of wound healing, compounded by poor angiogenesis that restricts the delivery of vital oxygen and nutrients required for cell proliferation and tissue repair (5). Unlike acute wounds that proceed through well-orchestrated inflammatory, proliferative, and remodeling phases, diabetic wounds become arrested in a prolonged inflammatory phase that prevents effective healing (6).

In recent years, the nucleotide-binding oligomerization domain-like receptor family pyrin domain-containing 3 (NLRP3) inflammasome has emerged as a critical player in the pathogenesis of diabetic complications, including wound chronicity (7). The NLRP3 inflammasome is a multiprotein complex that serves as a crucial component of the innate immune system, detecting various pathogen-associated molecular patterns (PAMPs) and damage-associated molecular pat-terns (DAMPs) (8). Upon activation, the NLRP3 inflammasome cleaves pro-caspase-1 to its active form, which subsequently processes pro-interleukin-1*β* (pro-IL-1*β*) and pro-IL-18 into their mature, biologically active forms (9). Additionally, caspase-1 mediates the cleavage of gasdermin D (GSDMD), leading to pyroptosis, a pro-inflammatory form of programmed cell death (10).

Amid these complex cellular and vascular impairments, Macrophages play a pivotal role in orchestrating the wound healing response through their remarkable phenotypic plasticity (11). In healthy wound healing, pro-inflammatory M1 macrophages predominate in the early inflammatory phase, efficiently clearing pathogens and cellular debris, before transitioning to anti-inflammatory M2 macrophages that promote tissue repair, angiogenesis, and extracellular matrix remodeling (12). However, in diabetic wounds, this temporal polarization pattern is disrupted, with persistent M1 macrophage accumulation and impaired M2 polarization contributing to chronic inflammation and healing failure (13).

To construct a comprehensive synthesis of these interrelated pathways, a structured literature search was conducted across PubMed, Scopus, and Web of Science databases for English-language publications up to 2026. Search strategies incorporated combination terms including “NLRP3 inflammasome”, “diabetic foot ulcer”, “macrophage polarization”, “NETosis”, and “pyroptosis”. Peer-reviewed primary research articles, mechanistic studies, and relevant clinical literature were prioritized to evaluate molecular drivers, temporal cellular dynamics, and emerging therapeutic interventions.

In this review, we synthesize current insights into the hyperinflammatory cascade driving diabetic wound impairment, with a primary focus on the activation and regulation of the NLRP3 inflammasome. Rather than treating these pathways in isolation, we examine how metabolic stress triggers NLRP3 signaling, explore its reciprocal crosstalk with macrophage polarization dynamics and NETosis, and evaluate emerging NLRP3-targeted therapeutic strategies that aim to reprogram the diabetic wound microenvironment toward effective resolution.

## 2 The NLRP3 inflammasome: structure and activation mechanisms

### 2.1 Inflammasome architecture and assembly

The NLRP3 inflammasome belongs to the NOD-like receptor (NLR) family of pattern recognition receptors and represents one of the most extensively studied inflammasome complexes (14). The core components of the NLRP3 inflammasome include the sensor protein NLRP3, the adaptor protein apoptosis-associated speck-like protein containing a CARD domain (ASC), and the effector protein caspase-1 (15). NLRP3 itself consists of three functional domains: an N-terminal pyrin domain (PYD) for homotypic protein interactions, a central nucleotide-binding and oligomerization domain (NACHT) with essential ATPase activity required for self-assembly, and a C-terminal leucine-rich repeat (LRR) domain that maintains the receptor in an autoinhibited conformation under basal conditions (16).

Inflammasome assembly is a highly regulated process that occurs through a canonical two-step mechanism involving priming and activation. The priming signal (Signal 1), typically provided by NF-κB-activating stimuli such as lipopolysaccharide (LPS) or tumor necrosis factor-α (TNF-α), induces the transcriptional upregulation of NLRP3, pro-IL-1β, and pro-IL-18 (17). While also licensing NLRP3 through critical post-translational modifications, such as deubiquitination. The subsequent activation signal (Signal 2) triggered by intracellular cellular stress events including K^+^ efflux, mitochondrial ROS release, or lysosomal disruption induces a conformational change in NLRP3 that exposes its NACHT domain, initiating self-oligomerization. This enables the PYD domain of NLRP3 to recruit ASC via homotypic PYD–PYD interactions. ASC then polymerizes into macromolecular filaments that aggregate into a single micrometer-scale ASC speck, bridging NLRP3 to pro-caspase-1 through caspase recruitment domain (CARD)–CARD interactions (18). This macromolecular platform facilitates the autoproteolytic cleavage of pro-caspase-1 into active caspase-1, which ultimately processes pro-IL-1β and pro-IL-18 into their mature secreted forms, alongside cleaving Gasdermin-D (GSDMD) to trigger pyroptotic cell death.

### 2.2 Triggers for NLRP3 inflammasome activation in diabetic wound

NLRP3 inflammasome activation is a multifaceted process that can be triggered by external and internal factors such as endogenous stress signals, pathogens, and a diverse array of stimuli in the microenvironment of diabetic wounds, persistent metabolic derangements specifically hyperglycemia, advanced glycation end-products (AGEs), and elevated free fatty acids act as primary upstream initiators. These metabolic stressors induce intracellular alterations, including excessive mitochondrial reactive oxygen species (ROS) generation, lysosomal destabilization, endoplasmic reticulum (ER) stress, and sustained K^+^ efflux, which collectively contribute to the chronic inflammatory state observed in diabetic wounds (19).

#### 2.2.1 Activation due to hyperglycemia

**Figure 1:**
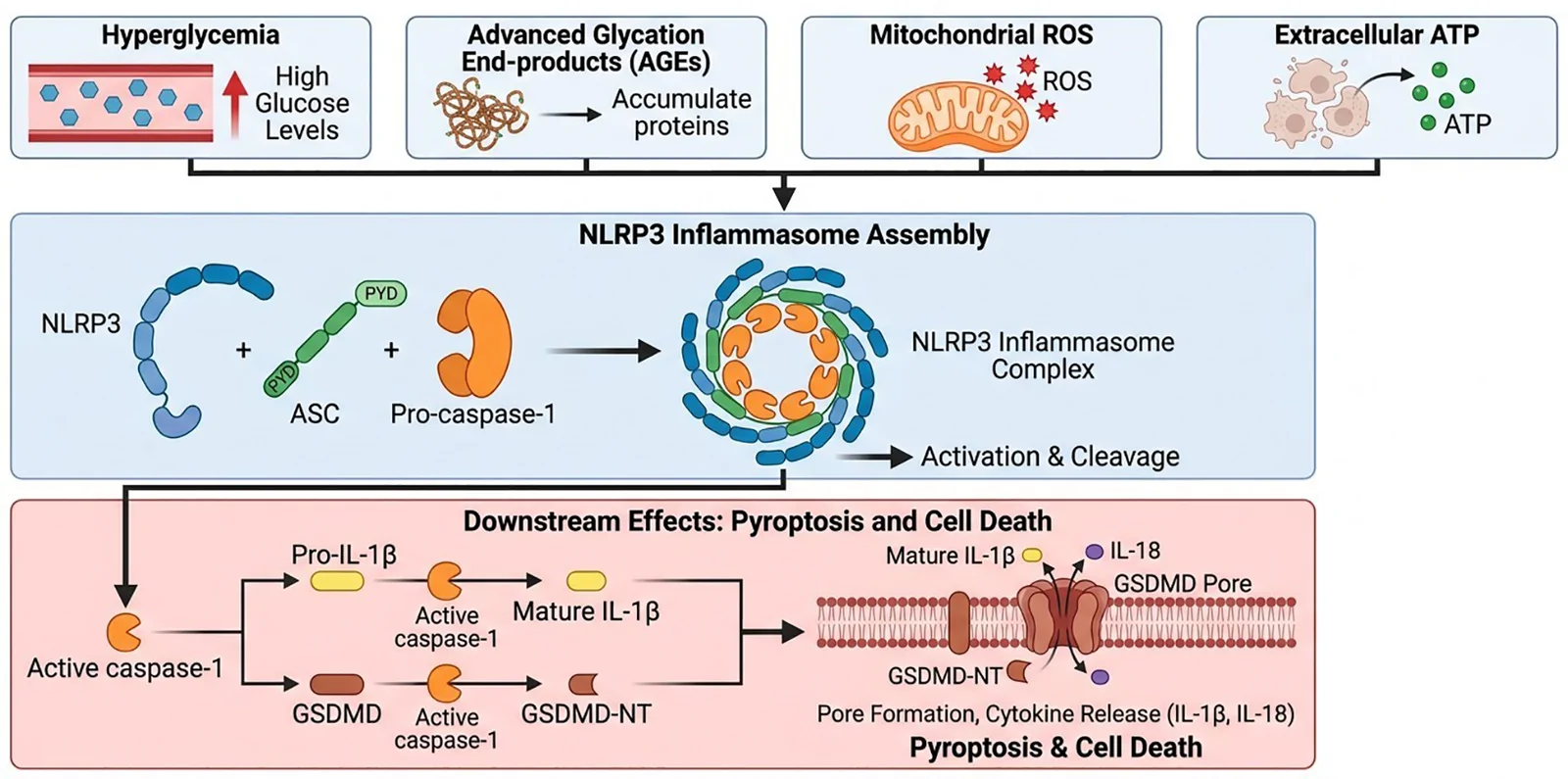
NLRP3 inflammasome activation pathway in diabetic wound context. The diagram illustrates the molecular cascade from diabetes-associated triggers (hyperglycemia, AGEs, mitochondrial ROS, extracellular ATP) to NLRP3 inflammasome assembly and down-stream pyroptotic effects. Key components include the sensor protein NLRP3, adaptor ASC, and effector caspase-1, which upon activation cleave pro-IL-1*β* and GSDMD, leading to pore formation, cytokine release, and inflammatory cell death.

Hyperglycemia represents a primary driver of inflammasome activation through several interconnected mechanisms. Elevated glucose levels promote the generation of mitochondrial reactive oxygen species (ROS), which directly activate NLRP3 through thioredoxin-interacting protein (TXNIP) dissociation and subsequent binding to NLRP3 (20). Zhou et al. demonstrated that mitochondrial dysfunction and the resulting ROS production are essential for NLRP3 inflammasome activation, with mitophagy blockade leading to accumulation of damaged, ROS-generating mitochondria and enhanced inflammasome activation (21) (**Figure 1**).

#### 2.2.2 Role of AGEs in triggering NLRP3 activation

Advanced glycation end-products (AGEs), which accumulate in diabetic tissues due to non-enzymatic glycation of proteins and lipids, represent another major trigger for NLRP3 activation (22). Specifically, the interaction of AGEs with their transmembrane receptor, RAGE, activates membrane-bound NADPH oxidase (NOX) complexes and downstream mitogen-activated protein kinase (MAPK) pathways including p38 and ERK1/2. This cascade induces sustained intracellular reactive oxygen species (ROS) production and activates the NF-κB transcription factor, which upregulates the priming expressions of NLRP3 and pro-IL-1β. Furthermore, AGE–RAGE signaling triggers intracellular calcium influx and mitochondrial dysfunction, which serve as crucial secondary signals driving NACHT domain oligomerization and canonical NLRP3 inflammasome assembly (23). In a streptozotocin-induced diabetic male C57BL/6 mouse model, Wan et al. demonstrated that the AGEs/ROS/NLRP3 inflammasome axis contributes to delayed corneal wound healing, with AGE accumulation promoting oxidative stress and subsequent inflammasome activation (24).

#### 2.2.3 Other triggers of NLRP3 inflammasome activation

Other triggers relevant to diabetic wound chronicity include extracellular ATP released from damaged cells, which activates the P2X7 receptor and triggers potassium efflux (25); cholesterol crystals and other particulate matter that cause lysosomal destabilization (26); and neutrophil extracellular traps (NETs) that amplify inflammatory signaling (27). The convergence of these various activation signals in the diabetic wound microenvironment creates a milieu conducive to sustained inflammasome activity.

## 3 Pyroptosis: a pro-inflammatory cell death pathway

### 3.1 Mechanisms of gasdermin D-mediated pyroptosis

Pyroptosis is an inflammatory form of programmed cell death mediated by gasdermin family proteins, with GSDMD serving as the primary executioner of inflammasome-induced pyroptosis (28). Upon caspase-1 cleavage, the N-terminal domain of GSDMD (GSDMD-NT) is released and translocates to the plasma membrane, where it oligomerizes to form large pores with an inner diameter of approximately 10–14 nm as measured by negative-stain electron microscopy (29). These pores facilitate the release of mature IL-1β and IL-18, as well as other intracellular contents including lactate dehydrogenase (LDH) and alarmins such as high-mobility group box 1 (HMGB1) (30).

The pore formation by GSDMD-NT disrupts ionic gradients across the plasma membrane, leading to cell swelling and eventual membrane rupture (31). Importantly, while pyroptosis results in cell death, the highly inflammatory nature of this process distinguishes it from immunologically silent forms of programmed cell death such as apoptosis. Specifically, the massive release of key pro-inflammatory cytokines predominantly mature IL-1β and IL-18 and intracellular alarmins directly suppresses local anti-inflammatory mediators (such as IL-10 and TGF-β1), thereby creating a severe cytokine imbalance that amplifies both local tissue destruction and systemic inflammatory responses (32).

### 3.2 Role of pyroptosis in diabetic wound chronicity

The contribution of pyroptosis to diabetic wound healing impairment has gained increasing recognition (33). Multiple studies have demonstrated elevated pyroptosis in diabetic wound tis-sues, characterized by increased expression of NLRP3, Caspase-1 activation, cleaved GSDMD, and mature IL-1β (34). Dai et al. reported that ROS-activated NLRP3 inflammasome initiates inflammation in delayed wound healing in diabetic rats, with pyroptosis contributing to the inflammatory microenvironment (19).

In diabetic mouse models (including Nlrp3 −/−, Casp1 −/−, and Gsdmd −/− strain variants) and primary neutrophil cultures, Yang et al. provided direct evidence for the role of pyroptosis in diabetic wound healing, demonstrating that disulfiram accelerates wound closure by blocking neutrophil extracellular trap (NET) formation via suppression of the NLRP3/caspase-1/GSDMD axis (35). This study highlighted the connection between pyroptosis and NETosis, revealing that GSDMD-mediated pore formation is required for NET release and that inhibition of this pathway can improve healing outcomes.

The pyroptosis of macrophages in diabetic wounds is particularly detrimental, as it eliminates key cells required for orchestrating the healing response while simultaneously releasing inflammatory mediators that perpetuate tissue damage (36). In a type 2 diabetic mouse model Wang et al. demonstrated that umbilical cord mesenchymal stem cell-derived apoptotic extracellular vesicles can ameliorate diabetic wound healing by inhibiting pro-healing M2 macrophages, highlighting the therapeutic potential of targeting this pathway (37).

## 4 Macrophage polarization in wound healing

### 4.1 The M1/M2 polarization paradigm

**Figure 2:**
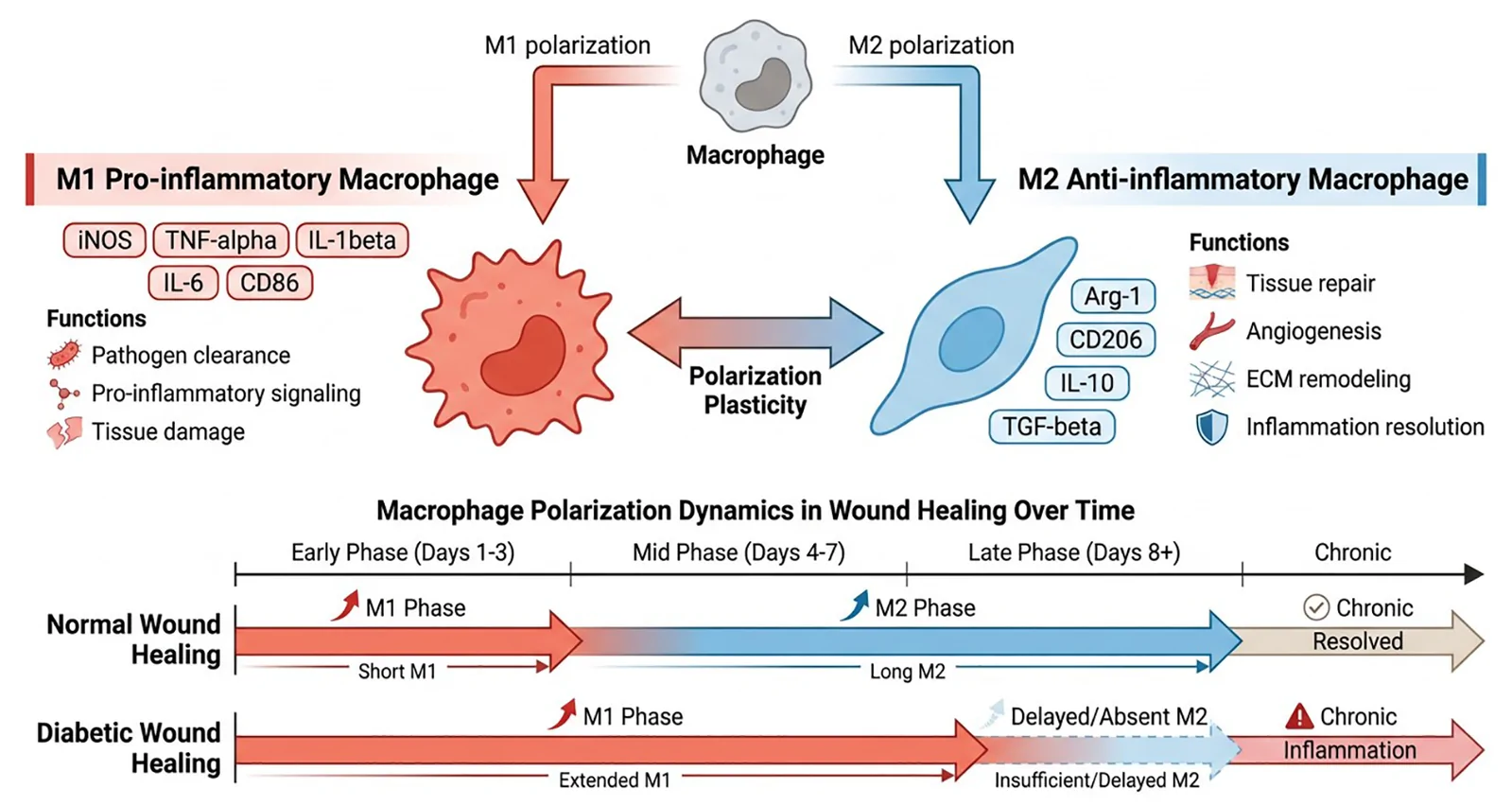
Macrophage polarization dynamics in diabetic wound healing. contrasts M1 pro-inflammatory macrophages (red, left; distinguished by a spiky, activated cellular contour) expressing iNOS, TNF- α, and IL-1β with M2 anti-inflammatory macrophages (blue, right; distinguished by an elongated, smooth cellular contour) expressing Arg-1, CD206, and IL-10. The lower timeline illustrates the temporal dynamics: normal wounds exhibit a brief M1 phase transitioning efficiently to M2, while diabetic wounds show prolonged M1 persistence with delayed or absent M2 transition, resulting in chronic inflammation and impaired healing. All cell phenotypes, molecular markers, and dynamic phases are further designated by explicit text labels.

Macrophages exhibit remarkable functional plasticity, adapting their phenotype in response to environmental cues (38). The M1/M2 polarization paradigm, while representing a simplified view of macrophage diversity, provides a useful framework for understanding macrophage function in wound healing (39). M1 macrophages are essential for pathogen clearance and initial inflammation but can cause tissue damage if present in excess. Classically activated M1 macrophages are induced by interferon-*γ* (IFN-*γ*) and lipopolysaccharide (LPS), producing pro-inflammatory cytokines including TNF-*α*, IL-1β, IL-6, and IL-12, and generating nitric oxide through inducible nitric oxide synthase (iNOS) (40). Alternatively activated M2 macrophages, induced by IL-4 and IL-13, express markers such as arginase-1 (Arg-1), mannose receptor (CD206), and chitinase-like proteins (41). M2 macrophages produce anti-inflammatory cytokines including IL-10 and transforming growth factor-*β* (TGF-*β*), promote angiogenesis, and facilitate extracellular matrix remodeling and tissue repair (42). The transition from M1 to M2 macrophage predominance is critical for the progression from the inflammatory to the proliferative phase of wound healing (43) (**Figure 2**).

### 4.2 Macrophage dysfunction in diabetic wounds

Diabetic wounds exhibit profound macrophage dysfunction characterized by several interconnected abnormalities (44). First, there is a delay in the recruitment of macrophages to the wound site, potentially due to impaired chemokine signaling and reduced neutrophil efferocytosis, leading to its dysfunction and prolonged inflammation (45). Second, the macrophages that do infiltrate diabetic wounds show a prolonged M1 phenotype with persistent expression of pro-inflammatory markers and impaired M2 polarization (46).

In both normoglycemic C57BL/6 and diabetic db/db mouse models, Guo et al. demonstrated that advanced glycation end-products (AGEs) induce autophagy, which impairs cutaneous wound healing by promoting macrophage polarization toward the pro-inflammatory M1 phenotype via IRF8 activation (47). This finding illustrates how the diabetic metabolic environment directly influences macrophage phenotype. Additionally, in diabetic mouse models and validated human diabetic wound tissues, Khanna et al. demonstrated that the diabetic microenvironment impairs macrophage efferocytosis the critical process of clearing apoptotic cells leading to an accumulation of apoptotic debris that further perpetuates chronic inflammation (48).

Recent single-cell transcriptomic analyses have refined and challenged the classical, simplistic M1/M2 binary paradigm in diabetic wound biology. Notably, Theocharidis et al. profiled over 174, 000 single cells from DFU patients and reported an unexpected spatially restricted enrichment of M1-like macrophages within the wound bed of healing ulcers, whereas non-healing ulcers exhibited higher proportions of M2-like macrophages alongside a specific subset of MMP-expressing fibroblasts (specifically enriched in MMP1, MMP3, and MMP10). While these findings appear at first glance to challenge the traditional concept that M1 persistence drives chronicity, they critically highlight that macrophage functionality is governed by strict spatiotemporal dynamics rather than static marker expression. In healing wounds, a robust, localized, and transient M1 response is functionally necessary for debris clearance, pathogen control, and initiation of early remodeling. Conversely, the accumulation of M2-skewed macrophages in non-healing wounds may represent a dysfunctional or stalled pro-resolving phenotype that fails to execute complete tissue repair (49). This underscores that diabetic wound chronicity stems not merely from an overabundance of M1 markers, but from a failure in the timely and spatially coordinated transition through macrophage functional states.

## 5 The inflammasome-macrophage axis in diabetic wounds

### 5.1 Bidirectional regulation between NLRP3 and macrophage phenotype

The relationship between NLRP3 inflammasome activation and macrophage polarization is bidirectional and mutually reinforcing (50). On one hand, NLRP3 activation and the resulting IL-1*β* production promote M1 macrophage polarization and inhibit M2 polarization (51). IL-1*β* activates NF-*κ*B signaling, which upregulates pro-inflammatory gene expression and maintains the M1 phenotype (52). The pyroptosis of M1 macrophages further releases damage-associated molecular patterns that amplify inflammasome activation in neighboring cells, creating a self-sustaining inflammatory cycle (53).

Conversely, M2 macrophages exhibit reduced NLRP3 inflammasome activation due to their anti-inflammatory phenotype and distinct metabolic programming (54). The transition from M1 to M2 polarization is associated with suppression of inflammasome components and restoration of tissue homeostasis (55). This reciprocal relationship suggests that interventions targeting the NLRP3 inflammasome may facilitate macrophage phenotype switching toward the pro-repair M2 state.

### 5.2 NETs and inflammasome amplification

Neutrophil extracellular traps (NETs) represent a crucial link between early inflammatory responses and sustained inflammasome activation in diabetic wounds (56). NETs are web-like structures composed of decondensed chromatin, histones, and granule proteins released by neutrophils during a specialized form of cell death termed NETosis (57). In diabetic wounds, NET formation is dysregulated, with excessive NET accumulation contributing to tissue damage and impaired healing (58).

The relationship between NETs and the NLRP3 inflammasome is bidirectional. On one hand, NET components including histones and DNA can activate the NLRP3 inflammasome in macrophages (59). Conversely, GSDMD-mediated pyroptosis has been implicated in promoting NET release (35). Huang et al. showed that milk fat globule-EGF factor 8 (MFG-E8) accelerates wound healing in diabetes by regulating the NLRP3 inflammasome-NET axis, demonstrating the therapeutic relevance of disrupting this pathway (27).

Li et al. demonstrated that suppressing NET formation by inhibiting NF-*κ*B enhances diabetic wound healing by blocking NLRP3 inflammasome activation, further supporting the importance of the NET-inflammasome axis in wound chronicity (60). These findings highlight multiple potential intervention points for breaking the cycle of sustained inflammation.

## 6 Therapeutic strategies targeting the inflammasome-macrophage axis

Given the central role of the NLRP3 inflammasome in driving chronic inflammation, targeting this signaling cascade and its cellular effectors has emerged as a novel therapeutic strategy for diabetic wound healing. An overview of these interventions encompassing pharmacological inhibitors, pyroptosis execution blockers, natural bioactives, cell-based therapies, and biomaterial delivery platforms is summarized in **Table 1**, detailing their molecular targets, model systems tested, and reported outcomes.

**Table 1:** Preclinical therapeutic strategies targeting the NLRP3 inflammasome–macrophage axis in diabetic wound healing.

Agent/intervention	Category	Primary molecular target	Model system tested	Key reported outcomes & mechanisms	Ref
MCC950	NLRP3 Inhibitor	Directly binds NACHT domain of NLRP3; blocks ATP hydrolysis	Preclinical DFU mouse model & primary murine macrophage co-cultures	Accelerates wound healing, promotes M1-to-M2 macrophage transition in an MDSC-dependent manner	(64)
MCC950	NLRP3 Inhibitor	Directly binds NACHT domain of NLRP3	Obese mouse model	Did not significantly alter wound healing and impaired healing in certain conditions, highlighting context-dependent effects	(65)
Glyburide	NLRP3 Inhibitor	NLRP3 inflammasome/K ATP ​ channels channels	Diabetic mouse model	Inhibits NLRP3 activation to expedite impaired fracture healing beyond its primary glucose-lowering effect	(68)
Disulfiram	GSDMD Inhibitor	Covalently modifies Cys191 in GSDMD pore-forming domain	Diabetic foot ulcer model	Prevents GSDMD oligomerization and pore formation, accelerating DFU healing by blocking NETosis via NLRP3/caspase-1/GSDMD axis	(35)
VX-765 (Belnacasan)	Caspase-1 Inhibitor	Caspase-1 active site	Preclinical inflammatory disease models	Prevents GSDMD cleavage and IL-1β/IL-18 maturation	(71)
Resveratrol	Natural Compound	Inflammasome/metabolic pathways	Diabetic mouse model	Facilitates macrophage polarization shift toward M2 and improves wound closure rates	(73)
Tanshinone IIA	Natural Compound	NLRP3 inflammasome pathway	Diabetic full-thickness wound rat model & *in vitro* human THP-1 cells	Suppresses macrophage NLRP3 activity, reduces IL-1β/IL-18 secretion, promotes M1-to-M2 transition, and accelerates tissue repair	(74)
Aloe vera Polysaccharides	Natural Compound	NLRP3 inflammasome activation	Diabetic wound models	Facilitates wound healing by promoting M2 macrophage polarization through NLRP3 inhibition	(76)
Melatonin-stimulated MSC Exosomes	Cell-based/Exosome	PTEN/AKT signaling axis	Diabetic wound models	Improves diabetic wound healing by regulating macrophage M1/M2 polarization balance	(79)
UC-MSC Apoptotic Vesicles	Cell-based/Exosome	Macrophage pyroptosis pathway	Type 2 diabetic mouse model	Ameliorates cutaneous wound healing by specifically inhibiting macrophage pyroptosis	(37)
Engineered Exosomes	Cell-based/Exosome	Targeted miRNAs/proteins against NLRP3 components	Diabetic wound microenvironment	Enables cell-free targeted modulation of macrophage phenotypes with higher specificity than systemic drugs	(80)
Silk Fibroin Dressings (A438079-loaded)	Biomaterial Delivery	P2X7 receptor (upstream NLRP3 activator)	Diabetic wound model	Combines biocompatible dressing with targeted P2X7 inhibition to modulate local inflammasome activity	(83)

### 6.1 Pharmacological inhibitors of NLRP3

The recognition of NLRP3 inflammasome activation as a key driver of diabetic wound chronicity has motivated the development of targeted therapeutic interventions (61). MCC950 (also known as CRID3 or CP-456, 773) is a potent and selective small-molecule inhibitor of the NLRP3 inflammasome that blocks both canonical and non-canonical inflammasome activation (62). MCC950 directly binds to the NACHT domain of NLRP3, preventing ATP hydrolysis and subsequent conformational changes required for inflammasome assembly (63).

Yan et al. demonstrated in a preclinical DFU mouse model and primary bone marrow-derived macrophage (BMDM) co-cultures that MCC950 significantly accelerates diabetic wound healing, shortening healing time and improving repair quality. Mechanistically, MCC950 promotes early recruitment of myeloid-derived suppressor cells (MDSCs) to the blood, spleen, and wound tissue, where they engage in cell crosstalk with macrophages to drive M2 macrophage polarization and downregulate pro-inflammatory gene expression (64). Notably, *in vivo* depletion of MDSCs eliminated the therapeutic benefits of MCC950, confirming that NLRP3 inhibition facilitates the transition toward pro-healing M2 macrophages in an MDSC-dependent manner and providing a precise mechanistic basis for its clinical potential. However, it should be noted that Lee et al. reported that MCC950 did not significantly alter wound healing in obese mice and even impaired healing in some conditions, suggesting that the effects may be context-dependent (65).

Glyburide, a sulfonylurea used clinically for type 2 diabetes treatment, has been identified as an NLRP3 inhibitor that blocks inflammasome activation at concentrations distinct from those required for ATP-sensitive potassium channel closure (66). While glyburide’s primary antidiabetic effect is through stimulating insulin secretion, its NLRP3 inhibitory properties may contribute to its therapeutic profile (67). Yang et al. demonstrated that glyburide expedites diabetic-induced impaired fracture healing by inhibiting the NLRP3 inflammasome, suggesting potential applications beyond glucose control (68).

### 6.2 Targeting pyroptosis execution

Inhibition of pyroptosis execution represents another therapeutic strategy for diabetic wound healing (69). Disulfiram, an FDA-approved drug for alcohol dependence, was identified as a GSDMD inhibitor that covalently modifies Cys191 in the pore-forming domain, preventing GS-DMD oligomerization and pore formation (70). Yang et al. demonstrated that disulfiram accelerates diabetic foot ulcer healing by blocking NET formation through suppression of the NLRP3/caspase-1/GSDMD pathway (35). This study established GSDMD as a viable therapeutic target and repurposed an existing drug for wound healing applications.

Other approaches to pyroptosis inhibition include caspase-1 inhibitors such as VX-765 (belnacasan), which prevents the processing of GSDMD and the maturation of IL-1*β* and IL-18 (71). While these agents have shown promise in preclinical models of inflammatory diseases, their application to diabetic wound healing requires further investigation.

### 6.3 Natural compounds and plant extracts

Beyond synthetic pharmaceuticals, natural bioactive compounds and plant extracts have emerged as promising immunomodulatory agents capable of dampening chronic inflammatory cascades, reducing oxidative stress, and promoting macrophage phenotype transitions in non-healing diabetic wounds (72).

Specifically, Ding et al. evaluated the therapeutic efficacy of resveratrol in a streptozotocin (STZ)-induced diabetic C57BL/6 mouse model over a 15-day period. Local peri-wound injection of resveratrol (10 µmol/L) significantly accelerated wound closure rates and enhanced tissue structural integrity by upregulating alpha-SMA and Collagen I expression. Furthermore, resveratrol markedly suppressed pro-inflammatory mediators (TNFα, iNOS, IL-1β) while driving M1-to-M2 macrophage polarization through elevated Arg-1 and CD206 expression via the PI3K/Akt pathway (73).

Tanshinone IIA, an active component of Salvia miltiorrhiza (Danshen), has been identified as an NLRP3 inflammasome inhibitor that accelerates diabetic wound healing (74). In a diabetic rat model with full-thickness dorsal skin wounds, validated *in vitro* using human THP-1 monocytes, Pan et al. demonstrated that Tanshinone IIA (Tan IIA) accelerates diabetic wound healing by targeting macrophage-mediated inflammation. Mechanistically, Tan IIA suppresses macrophage NLRP3 inflammasome activation, markedly decreasing the secretion of pro-inflammatory cytokines IL-1β and IL-18. This inflammasome inhibition drives the repolarization of M1 macrophages toward a pro-healing M2 phenotype, which significantly accelerates wound closure, enhances re-epithelialization, improves collagen organization, and stimulates local angiogenesis (74).

Aloe vera polysaccharides have been shown to facilitate diabetic wound healing by promoting macrophage M2 polarization through inhibition of NLRP3 inflammasome activation (75). This mechanism links the traditional use of aloevera in wound care to modern understanding of inflammasome biology.

### 6.4 Cell-based and exosome therapies

Mesenchymal stem cells (MSCs) and their secreted extracellular vesicles/exosomes (MSC-Exos) have emerged as promising therapeutic platforms for diabetic wound healing, operating through robust immunomodulatory, pro-angiogenic, and tissue-regenerative paracrine actions (76). Stem cell sources including bone marrow-derived MSCs (BM-MSCs), adipose-derived stem cells (ADSCs), and umbilical cord MSCs (UC-MSCs) exhibit multifaceted therapeutic strategies by dampening chronic inflammatory cascades, reducing oxidative stress, and promoting extracellular matrix remodeling in diabetic foot ulcers (DFUs). However, cell-free approaches utilizing MSC-derived exosomes are increasingly favored as they recapitulate these potent paracrine and immunomodulatory effects over the inflammasome-macrophage axis while overcoming ethical concerns, immunogenicity risks, and genomic instability associated with intact cell transplantation (77, 78). Mechanistically, using a streptozotocin (STZ)-treated diabetic mouse wound model alongside an air pouch model, Liu et al. demonstrated that exosomes derived from melatonin-pretreated MSCs (MT-Exo) significantly accelerate diabetic wound healing by promoting angiogenesis (α-SMA) and collagen synthesis (Collagen I and III). At the molecular level, MT-Exo suppressed pro-inflammatory factors (IL-β, TNF-α, iNOS) while elevating anti-inflammatory markers (IL-10, Arg-1) through increasing the ratio of M2 to M1 macrophage polarization via the PTEN/AKT signaling pathway (79).

Wang et al. showed that umbilical cord MSC-derived apoptotic extracellular vesicles ameliorate cutaneous wound healing in type 2 diabetic mice via macrophage pyroptosis inhibition (37). This study revealed that apoptotic vesicles from stem cells can specifically target pyroptotic pathways in macrophages, providing a novel mechanism for cell-free therapy.

Engineered exosomes designed to deliver specific miRNAs or proteins that target inflammasome components represent an emerging frontier in diabetic wound therapy (80). The ability to specifically target macrophages and modulate their phenotype through exosome-mediated delivery offers distinct therapeutic advantages over systemic drug administration, paving the way for targeted immunomodulatory interventions in chronic non-healing wounds.

### 6.5 Biomaterial-based delivery systems

Advanced biomaterials can be engineered to locally deliver inflammasome modulators to the wound site, maintaining therapeutic concentrations while minimizing systemic side effects (81). Hydrogels with enzyme-responsive degradation properties can release therapeutic agents in response to elevated matrix metalloproteinase activity characteristic of chronic wounds (82).

**Figure 3:**
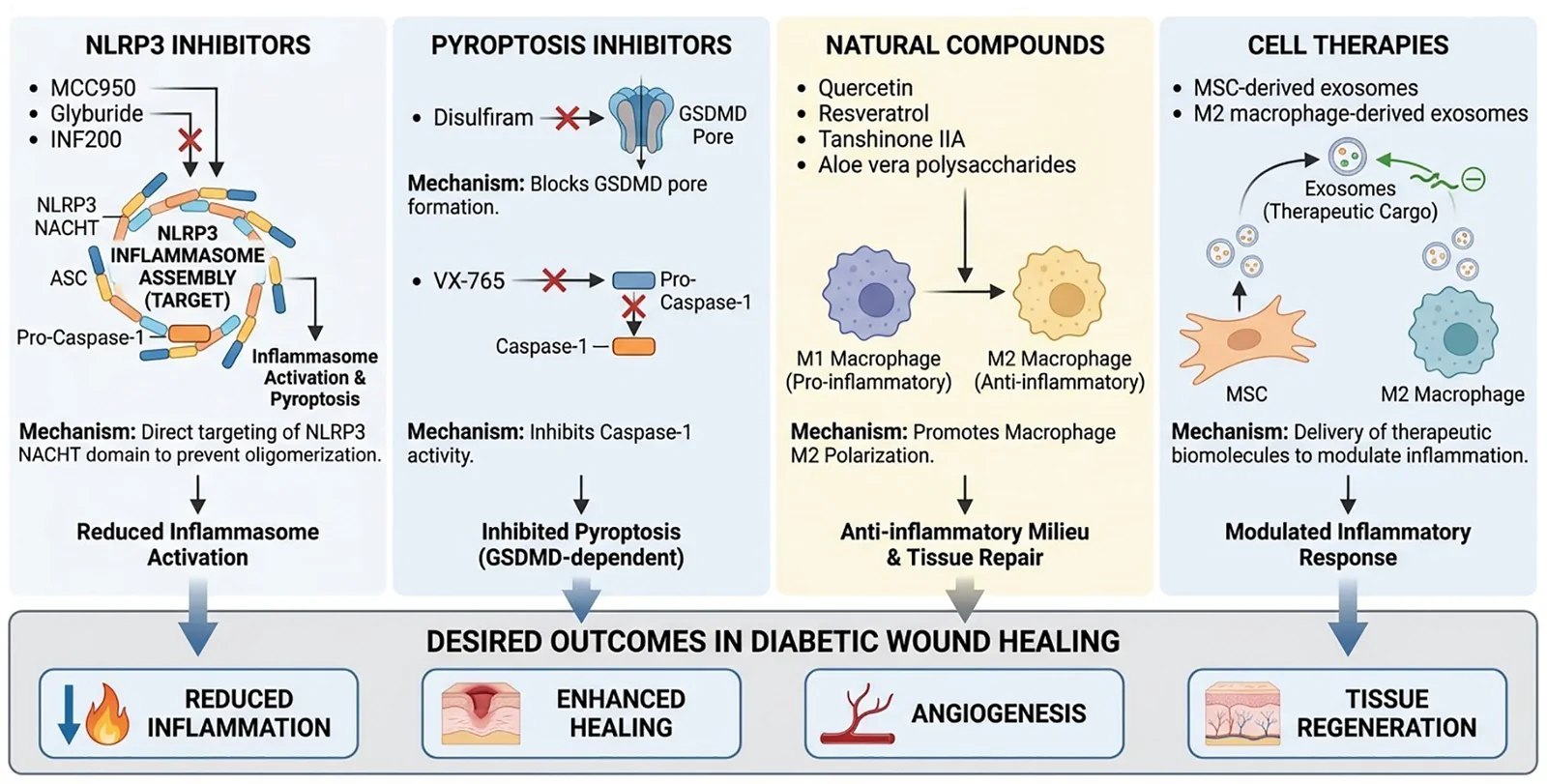
Therapeutic intervention strategies targeting the NLRP3 inflammasome-macrophage axis in diabetic wound healing. The diagram categorizes current therapeutic approaches into four main strategies: (1) NLRP3 inhibitors (MCC950, glyburide) targeting the sensor protein, (2) pyroptosis inhibitors (disulfiram, VX-765) blocking downstream execution, (3) natural compounds (quercetin, resveratrol, tanshinone IIA) promoting M2 polarization, and (4) cell-based therapies (MSC-derived exosomes) delivering therapeutic cargo. All pathways converge on the desired outcomes of reduced inflammation, enhanced healing, angiogenesis, and tissue regeneration.

Yaron et al. developed silk fibroin wound dressings loaded with the P2X7 inhibitor A438079 for inflammasome modulation in diabetic wound healing (83). This approach combines the bio-compatibility of silk fibroin with targeted inhibition of P2X7 receptor-mediated NLRP3 activation. Similarly, nanoparticle-based delivery systems can enhance the bioavailability of natural compounds and enable sustained release at the wound site (84) (**Figure 3**).

## 7 Future perspectives and clinical translation

The evidence reviewed here establishes the NLRP3 inflammasome as a central node in the network of pathological processes that contribute to diabetic wound chronicity (85). The con-vergence of multiple diabetes-associated triggers hyperglycemia-induced mitochondrial dysfunction, AGE accumulation, oxidative stress, and NET formation creates conditions favoring sustained inflammasome activation in the diabetic wound microenvironment (86). This activation initiates a cascade of events including caspase-1 activation, IL-1β and IL-18 maturation, and GSDMD-mediated pyroptosis that perpetuates inflammation and impairs healing progression.

The bidirectional relationship between inflammasome activation and macrophage polariza-tion creates a self-sustaining cycle of chronic inflammation (87). NLRP3 activation promotes M1 macrophage polarization while inhibiting the transition to M2 phenotype, and the resulting pro-inflammatory environment further amplifies inflammasome signaling (88). Breaking this cycle through targeted interventions represents a promising therapeutic strategy.

Despite significant progress in understanding the role of NLRP3 inflammasome in diabetic wound healing, several challenges remain (89). First, the temporal dynamics of inflammasome activation during the wound healing process are incompletely understood. Sustained activation is highly detrimental because it traps the wound in a chronic inflammatory state by driving persistent cytokine release and execution of pyroptosis; however, physiological or transient inflammasome activity during the initial stage is necessary for host defense, clearing tissue debris, and priming early immune responses (90). Determining the optimal timing and duration of inflammasome inhibition will be crucial for therapeutic development.

Second, the cell-type-specific roles of the inflammasome in wound healing require further investigation (91). While macrophages are clearly important targets, keratinocytes, fibroblasts, and endothelial cells also express inflammasome components and may respond differently to activation signals (92). Clarifying whether cell-based therapies promote downregulation of the M1 phenotype, facilitate switching toward the regenerative M2 axis, or upregulate anti-inflammatory cytokines will be key to achieving optimal therapeutic outcomes.

Third, the translation of preclinical findings to clinical practice faces significant hurdles (93). Creating a stable diabetic rodent wound model requires precise technical acumen and rigorous validation parameters, such as confirming persistent hyperglycemia via STZ or genetic models prior to full-thickness wound induction. However, many studies use STZ-induced mice, which model type 1 diabetes rather than the more prevalent human type 2 diabetes (94). Furthermore, issues surrounding low reproducibility, variable drug safety/efficacy, and inter-species differences in wound healing pose major translational barriers. To bridge this gap, human induced pluripotent stem cell (iPSC)-based *in vitro* disease modeling offers a powerful platform to study human inflammasome biology in petriplate models, reducing the bench-to-bedside translational gap.

The heterogeneity of DFUs suggests that personalized approaches to inflammasome-targeted therapy may be necessary (95). Biomarkers of inflammasome activation, such as IL-1β and IL-18 levels in wound fluid, could identify patients most likely to benefit from specific interventions (96). Combining inflammasome modulation with other targeted therapies based on individual wound characteristics may optimize outcomes.

Advances in tissue engineering and regenerative medicine offer opportunities to integrate inflammasome modulation with scaffold-based approaches (97). Prospective biological dressings, such as human amniotic membrane applications, as well as genetically engineered biomaterials, provide promising matrices for wound repair. Furthermore, smart biomaterials that sense the inflammatory microenvironment and release therapeutic agents in response may provide personalized, adaptive therapy (98). The combination of metabolic intervention to address underlying diabetes with local wound therapy targeting the inflammasome-macrophage axis represents a comprehensive approach to diabetic wound management.

## 8 Conclusion

The NLRP3 inflammasome serves as a central pathogenic node in diabetic wound chronicity, bridging intracellular metabolic stress with sustained extracellular tissue hyperinflammation. Rather than operating in isolation, persistent inflammasome signaling reinforces an inflammatory feedback loop that halts the resolution phase of healing and locks macrophages in a detrimental pro-inflammatory state. Targeting the NLRP3 pathway whether through direct pharmacological inhibition, natural bioactives, or exosome-based therapies represents a transformative strategy to restore the healing cascade. Combining these molecular interventions with advanced biomaterial delivery platforms holds significant promise for achieving sustained, localized therapeutic efficacy in diabetic foot ulcers.

However, translating these promising preclinical discoveries into successful bedside clinical applications faces several significant translational hurdles. A primary obstacle is the stark discrepancy between animal models (which typically involve acute, surgically induced wounds in young, genetically homogeneous rodents) and human diabetic foot ulcers (which present with complex, chronic comorbidities, altered biomechanics, and distinct species-specific immune dynamics). Furthermore, systemic administration of small-molecule inflammasome inhibitors often suffers from poor bioavailability, off-target effects, and potential immunosuppressive toxicity. While cell-free therapies (such as exosome-based interventions) and biomaterial platforms offer localized delivery, they face rigorous clinical translation challenges, including batch-to-batch heterogeneity, large-scale manufacturing standardization, long-term biocompatibility, and strict regulatory approvals.

To translate these preclinical insights into clinical practice, future studies must prioritize validating these target pathways in human diabetic wound tissues, clarifying cell-type-specific mechanics, and resolving these translational barriers. Moving from symptomatic management to precise immunomodulatory targeted therapies will be essential to reduce the global burden of diabetic foot ulcers.
